# Impaired mitophagy induces antimicrobial responses in macrophages infected with *Mycobacterium tuberculosis*

**DOI:** 10.1186/s13578-023-01107-2

**Published:** 2023-08-30

**Authors:** Junghwan Lee, Seong-Ahn Lee, Sang-Hun Son, Ji-Ae Choi, Tam Doan Nguyen, Jaewhan Kim, Doyi Son, Chang-Hwa Song

**Affiliations:** 1 Department of Microbiology, Department of Medical Science, College of Medicine, Chungnam National University, 266 Munhwa‑ro, Jung‑gu, Daejeon, 35015 South Korea; 2 Department of Medical Science, College of Medicine, Chungnam National University, 266 Munhwa-ro, Jung-gu, Daejeon, 35015 South Korea; 3 Translational Immunology Institute, Chungnam National University, Daejeon, 34134 South Korea

**Keywords:** Macrophage, Mitophagy, *Mycobacterium tuberculosis*, BNIP3, mROS

## Abstract

**Background:**

Mitophagy, mitochondrial selective autophagy, plays a pivotal role in the maintenance of cellular homeostasis in response to cellular stress. However, the role of mitophagy in macrophages during infection has not been elucidated. To determine whether mitophagy regulates intracellular pathogen survival, macrophages were infected with *Mycobacterium tuberculosis* (Mtb), an intracellular bacterium.

**Results:**

We showed that Mtb-infected macrophages induced mitophagy through BCL2/adenovirus E1B 19 kDa protein-interacting protein 3 (BNIP3) activation. In contrast, BNIP3-deficient macrophages failed to induce mitophagy, resulting in reduced mitochondrial membrane potential in response to Mtb infection. Moreover, the accumulation of damaged mitochondria due to BNIP3 deficiency generated higher levels of mitochondrial reactive oxygen species (mROS) compared to the control, suppressing the intracellular survival of Mtb. We observed that siBNIP3 suppressed intracellular Mtb in mice lungs.

**Conclusion:**

We found that BNIP3 plays a critical role in the regulation of mitophagy during Mtb infection. The inhibition of mitophagy suppresses Mtb growth in macrophages through increased mROS production. Therefore, BNIP3 might be a novel therapeutic target for tuberculosis treatment.

**Supplementary Information:**

The online version contains supplementary material available at 10.1186/s13578-023-01107-2.

## Background

*Mycobacterium tuberculosis* (Mtb), a pathogen that causes tuberculosis (TB), is aerosolized and inhaled into the lungs [1]. In the lungs, Mtb is phagocytosed by alveolar macrophages and then killed in phagolysosomes, organelles produced by the fusion of phagosomes and lysosomes [2, 3]. However, virulent Mtb can escape the phagolysosome by forming pores in the phagosome, and surviving in host cells [1, 4]. It has been reported that intracellular Mtb disrupts mitochondrial dynamics and function by perturbing mitochondrial membrane potential (MMP) and altering reactive oxygen species (ROS) production, leading to mitochondrial dysfunction [5].

Mitochondria are essential for various biological functions, including energy production, the regulation of cell death, and the production of ROS [6]. ROS are messengers of cell signal transduction, which could also be used to remove pathogens from immune cells [7]. However, excessive generation of ROS triggers oxidative damage to mitochondria, leading to alterations in mitochondrial dynamics [8] and, ultimately, the removal of damaged mitochondria via mitophagy [8]. Mitophagy, a mitochondria-specific selective autophagy, is important for the maintenance of mitochondrial quality control in cells [9, 10]. Bcl2/adenovirus E1B 19 kDa protein-interacting protein 3 (BNIP3), one of the mitophagy-related proteins, binds directly to microtubule-associated protein 1A/1B-light chain 3 (LC3) to form an autophagosome, which then fuses with a lysosome, resulting in mitochondrial degradation [8, 11–13]. In response to mitochondrial damage due to infection or environmental stress, BNIP3 is expressed and localized to the mitochondria [14]. Several reports indicate that BNIP3-dependent mitophagy inhibits the production of ROS, leading to the survival of cardiomyocytes, tumor cells, and hepatocytes [15–17].

Mitochondria are important for the control of pathogen survival. A previous study revealed that dengue virus (DENV) infection interfered with mitochondrial dynamics by inducing mitochondrial fission and, as a result, supporting DENV replication [18]. Another virus, the human hepatitis B virus (HBV), also induces mitochondrial fission and increases mitophagy, promoting cell survival and HBV replication [19]. Furthermore, mitophagy-mediated decreased mitochondrial ROS (mROS) levels suppress NLRP3 inflammasome activation, leading to the alleviation of influenza virus-induced inflammatory lesions [20]. Alterations in mitochondrial dynamics are also critical for controlling pathogenic bacterial infections. An earlier report suggested that mitochondrial fission decreases the intracellular survival of *Listeria monocytogenes* (*L. monocytogenes*) [21], while its toxin Listeriolysin O is involved in mitophagy induction [22]. The inhibition of *L. monocytogenes*-induced mitophagy activates the production of mROS, leading to the suppression of *L. monocytogenes* [22]. Another study suggested that *Vibrio splendidus* increased MMP to generate high levels of mROS, inducing mitochondrial injury, and that the damaged mitochondria are eliminated by BNIP3-mediated mitophagy, suppressing ROS-induced cell death [23]. Therefore, mitochondrial dynamics and mitophagy are important for pathogen survival.

Our previous study suggested that Mtb-induced mitochondrial dysfunction plays a critical role in the survival of mycobacteria in macrophages [24]. Several Mtb antigens, such as the early secretory antigenic target 6 kDa (ESAT-6) and 38 kDa antigen, are involved in ROS production, leading to macrophage apoptosis [25, 26]. Another group proposed that Mtb induced ultrastructural changes in mitochondria, resulting in mitochondrial dysfunction [27]. These reports suggest that not only Mtb but also secreted Mtb antigens trigger mitochondrial dysfunction through ROS generation. However, it is not well known whether Mtb-induced mitochondrial dysfunction affects the activation of mitophagy. In this study, we investigated the mechanism through which BNIP3 regulates mitophagy during Mtb infection.

## Results

### Mtb infection induces expression of BNIP3 in macrophages

**Fig. 1:**
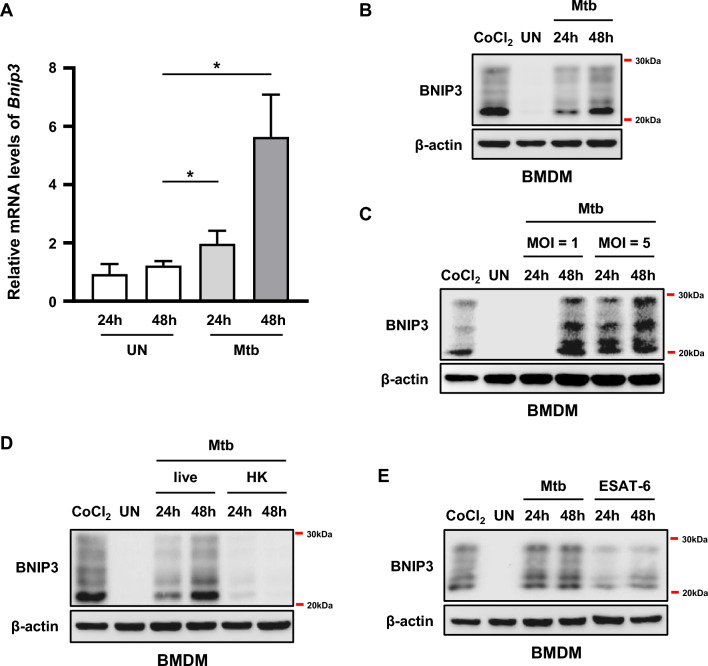
Mtb infection induces BNIP3 expression in macrophages. **A**, **B** Bone marrow-derived macrophages (BMDMs) were infected with Mtb at a multiplicity of infection (MOI) of 1 for 24 and 48 h. **A** *Bnip3* mRNA was analyzed using quantitative real-time PCR. **B** Levels of BNIP3 at 24 and 48 h post infection were evaluated using western blotting. **C** BMDMs were infected with Mtb (MOI = 1 or 5) for 24 and 48 h. **D** BMDMs were infected with live or heat-killed (HK) Mtb (MOI = 1) for 24 and 48 h. **E** BMDMs were treated with ESAT-6 (10 μg/ml) or Mtb (MOI = 1) for 24 and 48 h, and expression of BNIP3 was evaluated using western blotting. BMDMs were treated with CoCl_2_ (200 μM) for 24 h and used as a positive control. β-actin was used as a loading control. Data are representatives from at least three independent experiments (mean ± SD of *n* = 4 in A); *p < 0.05

First, to evaluate whether Mtb affected BNIP3 expression, we examined the mRNA levels of *Bnip3* in Mtb-infected macrophages. The mRNA expression of *Bnip3* was significantly induced in bone marrow-derived macrophages (BMDMs) infected with Mtb 48 h post infection (Fig. 1A). Next, we determined the effect of Mtb infection on BNIP3 protein levels. Cobalt(II) chloride (CoCl_2_), an established chemical inducer of hypoxia-like responses, was used as the positive control in these experiments. Our results showed that the protein levels of BNIP3 were increased in Mtb-infected macrophages between 24 and 48 h post infection (Fig. 1B). We further confirmed that BNIP3 was induced by Mtb infection by evaluating BNIP3 expression in a multiplicity of infection (MOI)-dependent manner. As expected, there was a direct correlation between BNIP3 expression in macrophages and MOI (Fig. 1C). To determine whether inactivated Mtb could induce BNIP3 in macrophages, BMDMs were infected with live or heat-killed Mtb for 48 h. The expression of BNIP3 was increased by live Mtb but not by heat-killed Mtb (Fig. 1D). Next, we treated macrophages with live Mtb, as well as ESAT-6, a well-known antigen secreted by live Mtb. Our results showed that, similar to live Mtb, ESAT-6 induced BNIP3 protein expression in macrophages (Fig. 1E), further indicating that live Mtb infection upregulated the expression of BNIP3 in macrophages.

### BNIP3 is induced through HIF1α activation in Mtb-infected macrophages

**Fig. 2:**
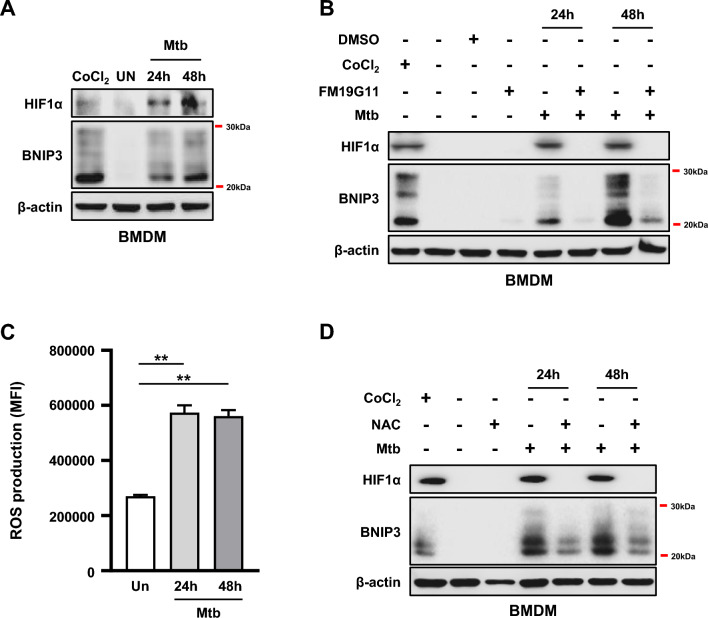
BNIP3 expression is induced via HIF1α activation in Mtb-infected macrophages. **A** BMDMs were infected with Mtb at an MOI of 1. The levels of HIF1α and BNIP3 were evaluated using western blotting. **B** BMDMs were pretreated with FM19G11 (20 μM) for 1 h and then infected with Mtb (MOI = 1) for 3 h. Cell lysates were collected 24 and 48 h post infection, western blotting was performed, and blots were probed using antibodies against HIF1α, BNIP3, and β-actin. **C** BMDMs were infected with Mtb (MOI = 1) for 48 h, and the levels of ROS were measured using a DHE stain (*n* = 5 per group). **D** BMDMs were pretreated with the ROS scavenger NAC (20 mM) for 1 h and then infected with Mtb at an MOI of 1 for 24 and 48 h. BMDMs treated with CoCl_2_ (200 μM) for 24 h were used as the positive control. Protein levels were analyzed by western blotting. β-actin was used as a loading control. Representative blots of three independent experiments are shown. The data are presented as means ± SD of least three independent experiments; **p < 0.01

In hypoxia, BNIP3 is the target gene of hypoxia-inducible factor-1-alpha (HIF1α) [28, 29]; however, it is not clear whether BNIP3 is regulated by HIF1α during Mtb infection in the absence of hypoxia. We investigated the protein levels of HIF1α and BNIP3 in Mtb-infected macrophages, and our results showed that expression of HIF1α and BNIP3 increased in a time-dependent manner (Fig. 2A). Next, to investigate whether HIF1α is located upstream of BNIP3, we treated Mtb-infected macrophages with FM19G11, a specific inhibitor of HIF1α, and observed that BNIP3 expression was reduced in the presence of HIF1α inhibitor (Fig. 2B). These data indicated that in macrophages, Mtb induced BNIP3 expression via HIF1α. As the name implies, oxygen concentration is the primary regulator of HIF1α expression and function; however, it can also be activated by ROS [30]. To investigate whether Mtb infection induces ROS in macrophages, we measured ROS production, which was increased in Mtb-infected macrophages at 24 and 48 h (Fig. 2C). Furthermore, these increased levels of ROS were reduced by N-acetyl-L-cysteine (NAC), resulting in decreased HIF1α and BNIP3 expression (Fig. 2D). These results suggested that ROS generation in response to Mtb infection induced the activation of HIF1α-BNIP3 axis in macrophages.

### Mitophagy inhibition accumulates mROS in BNIP3-deficient macrophages during Mtb infection

**Fig. 3:**
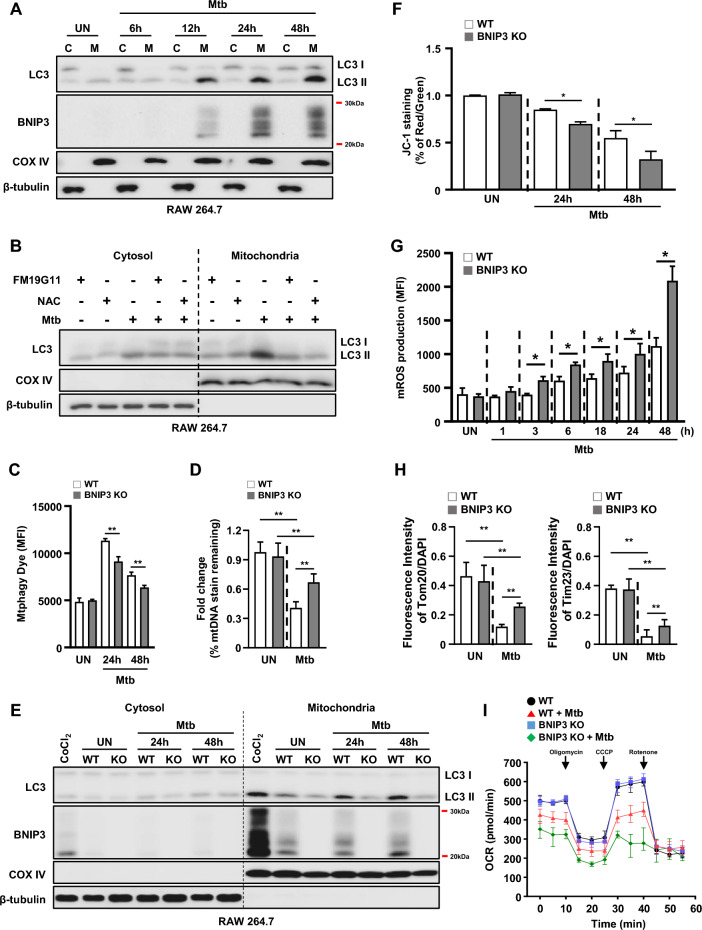
Mitophagy is regulated by BNIP3 in Mtb-infected macrophages. **A** RAW 264.7 cells were infected with Mtb at an MOI of 1. Western blot analysis was performed using mitochondrial and cytosolic fractions from Mtb-infected macrophages.** B** RAW 264.7 cells were pretreated with FM19G11 (20 μM) or NAC (20 mM) for 1 h and then infected with Mtb (MOI = 1) for 3 h. Cell lysates were collected 48 h post infection. **C** Mitophagy was measured in RAW 264.7 cells using a mitophagy detection kit (*n* = 5 per group). **D** The fluorescence intensity ratios of mitochondrial DNA in figure S3. The data are presented from at least three independent experiments (means ± SD of *n* = 8). **E** BNIP3-knockout (BNIP3-KO) RAW 264.7 cells were separated into cytosolic and mitochondrial fractions after Mtb infection for 48 h (MOI = 1). RAW 264.7 cells were treated with CoCl_2_ (200 μM) for 24 h. Protein levels of LC3 and BNIP3 were measured by western blotting. COX IV and β-tubulin were used as mitochondrial and cytosolic fraction loading controls, respectively. **F** Mitochondrial membrane potential (MMP) was measured in RAW 264.7 cells using JC-1 staining. Fluorescent intensity of JC-1 was detected at excitation wavelength 488 nm and emission wavelength 530 nm using flow cytometry (*n* = 4 per group). **G** Mtb-infected wild-type (WT) and BNIP3-KO RAW 264.7 cells were incubated with MitoSOX Red to measure mitochondrial ROS (mROS) levels. The mROS levels were analyzed using flow cytometry (*n* = 4 per group). **H** The fluorescence intensity ratios of Tom20 and Tim23 in figure S4. The data are presented from at least three independent experiments (means ± SD of *n* = 5). **I** Oxygen consumption rate (OCR) was measured after sequential treatment with oligomycin, carbonyl cyanide 3-chlorophenylhydrazone (CCCP), and rotenone (*n* = 5 per group). The data are presented as means ± SD of at least three independent experiments; *p < 0.05, **p < 0.01

It has been reported that activated BNIP3 is localized on the mitochondrial surface [14]. BNIP3 is a mitophagy receptor that interacts with LC3 to clear damaged mitochondria [8, 31, 32]. To confirm the localization of BNIP3 in response to Mtb infection, we analyzed the cytosolic and mitochondrial fractions of macrophages infected with Mtb. Our results showed that BNIP3 expression levels were increased in a time-dependent manner in response to Mtb infection (Fig. 3A). Moreover, the levels of lipidated LC3 (LC3-II) also increased in the mitochondrial fraction of Mtb-infected macrophages. However, treatment with FM19G11 and NAC reversed the increased expression of LC3-II during Mtb infection (Fig. 3B). Next, we examined whether mitophagy was affected in BNIP3-knockout (BNIP3-KO) RAW 264.7 macrophages in response to Mtb infection. We used Mtphagy Dye for the detection of mitophagy because it targets mitochondria and is useful in detecting mitochondrial acidification [33]. Mtb-induced mitophagy was also significantly decreased in BNIP3-KO macrophages compared to wild-type (WT) controls (Fig. 3C; Additional file 1: Fig. S1). In addition, increased mitophagy by Mtb was reduced during treatment with lysosomal inhibitor such as chloroquine or bafilomycin (Additional file 1: Fig. S2). Next, we measured mtDNA nucleoid as a secondary indicator of mitophagy using immunofluorescence [34]. The mtDNA was decreased in WT macrophages infected with Mtb compared to BNIP3-KO macrophages (Fig. 3D; Additional file 1: Fig. S3). Interestingly, the expression of LC3-II in the mitochondrial fraction of Mtb-infected macrophages was reduced in BNIP3-KO macrophages compared to the WT controls (Fig. 3E). These results suggest that BNIP3 is necessary for the induction of mitophagy in Mtb-infected macrophages. Disruption of mitochondrial homeostasis due to impaired mitophagy can lead to cellular damage, such as excessive ROS generation and MMP depolarization [35]. To investigate whether mitophagy inhibition exacerbated mitochondrial damage during Mtb infection, we measured MMP, mitophagy, and mROS levels in Mtb-infected BNIP3-KO cells. Our results showed that Mtb-induced MMP was reduced in BNIP3-KO macrophages in a time-dependent manner compared to the WT controls (Fig. 3F). As expected, the levels of mROS were increased at 48 h in Mtb-infected BNIP3-KO macrophages compared to those in the control group (Fig. 3G). Next, we measured the mitochondrial oxygen consumption rate (OCR) of the WT or BNIP3-KO RAW 264.7 cells at 48 h during Mtb infection. The basal OCR was decreased in Mtb-infected BNIP3-KO RAW 264.7 cells compared to Mtb-infected WT controls (Fig. 3I). Next, we analyzed the mitochondrial quantity using immunofluorescence. Mtb infection reduced the expression of mitochondria markers, such as Tom20 and Tim23, in macrophages (Fig. 3H; Additional file 1: Fig. S4). However, the expression of Tom20 and Tim23 in BNIP3-KO macrophages increased compared to the WT controls. These data suggest that Mtb causes mitochondrial dysfunction, but the clearance of damaged mitochondria is impaired because of the reduced mitophagy in BNIP3-KO RAW 264.7 cells. Thus, BNIP3 played an important role in Mtb-induced mitophagy in macrophages, while the inhibition of mitophagy aggravated mitochondrial impairment.

### The mROS accumulation due to mitophagy inhibition increases proinflammatory cytokine levels

**Fig. 4:**
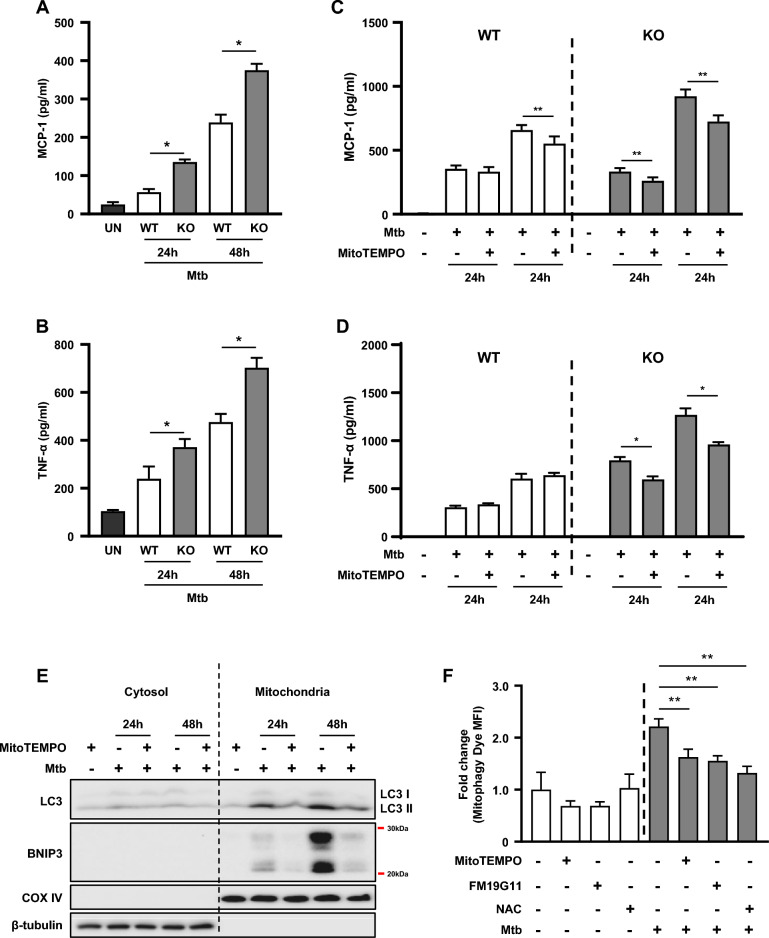
The accumulation of mROS due to mitophagy inhibition increases the levels of proinflammatory cytokines. **A**, **B** WT and BNIP3-KO RAW 264.7 cells were infected with Mtb for 48 h (MOI = 1). **C**, **D** WT and BNIP3-KO RAW 264.7 cells were treated with MitoTEMPO (100 μM), a mitochondrial ROS scavenger, and then infected with Mtb at an MOI of 1 for 24 and 48 h. The analysis of proinflammatory cytokine levels was performed in macrophages at 24 and 48 h post infection. **E** RAW 264.7 cells were treated with MitoTEMPO (100 μM) and then infected with Mtb at an MOI of 1. Western blot analysis was performed using mitochondrial and cytosolic fractions from Mtb-infected macrophages. **F** RAW 264.7 cells were treated with NAC (20 mM), FM19G11 (20 μM), or MitoTEMPO (100 μM) for 1 h and then infected with Mtb at an MOI of 1 for 48 h. Data are representatives from at least three independent experiments (mean ± SD of *n* = 4 per group in **A**, **B**, **F**; *n* = 6 per group in **C**, **D**); *p < 0.05, **p < 0.01

We showed that the inhibition of mitophagy via BNIP3 deficiency resulted in the accumulation of mROS (Fig. 3). Since ROS induce several immune responses, including the production of cytokines during infection [36], we examined the levels of proinflammatory cytokines in macrophages infected with Mtb. We found that the production of proinflammatory cytokines, including monocyte chemoattractant protein (MCP)-1 and tumor necrosis factor (TNF)-α, was increased during Mtb infection in BNIP3-KO macrophages compared to the control group (Fig. 4A, B). To further investigate the effect of mROS accumulation on the levels of proinflammatory cytokines during Mtb infection, the cells were treated with MitoTEMPO, a mitochondria-targeted antioxidant. MitoTEMPO decreased the secretion of MCP-1 and TNF-α by macrophages in response to Mtb infection (Fig. 4C, D), and this effect was observed at both the 24- and 48-h time points. In addition, MitoTEMPO reduced the levels of LC3-II, BNIP3, and mitophagy during Mtb infection (Fig. 4E, F). NAC and FM19G11 also decreased the mitophagy levels in Mtb-infected RAW 264.7 cells (Fig. 4F). These results suggest that the accumulation of mitochondrial superoxide through BNIP3 deficiency increased the levels of proinflammatory cytokines.

### BNIP3 inhibition suppresses the intracellular survival of Mtb in macrophages

**Fig. 5:**
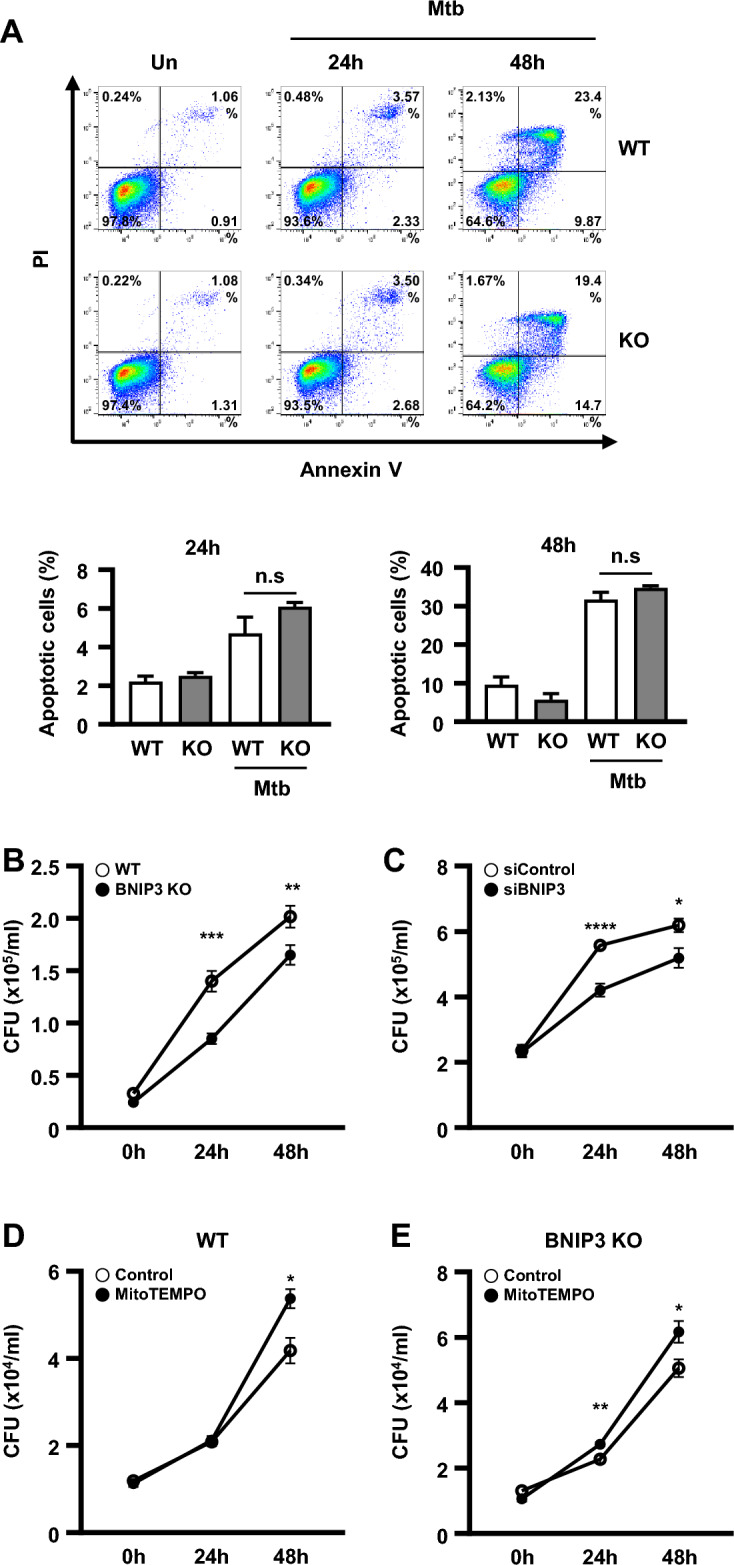
Mitophagy regulates the intracellular survival of Mtb through BNIP3. **A** The percentage of apoptotic cells was determined in BNIP3-KO RAW 264.7 cells infected with Mtb (MOI = 1) for 24 and 48 h (*n* = 3 per group). **B**, **C** BNIP3-KO RAW 264.7 cells and siRNA (siControl or siBNIP3)-treated BMDMs were infected with Mtb (MOI = 1) for 24 and 48 h, and the intracellular survival was determined by counting the number of colony-forming units (CFU) (*n* = 9 per group). **D**, **E** WT or BNIP3-KO cells were treated with MitoTEMPO (100 μM) and then infected with Mtb (MOI = 1) for 24 and 48 h (*n* = 9 per group). Intracellular survival analysis was performed as described in **B**, **C**. The data are presented as means ± SD of least three independent experiments; *p < 0.05, **p < 0.01, ***p < 0.001, ****p < 0.0001

BNIP3 contains an LC3-interacting region (LIR) motif as well as a Bcl-2 homology 3 (BH3) region, indicating its role in the induction of apoptosis [37, 38]. To confirm the involvement of BNIP3-mediated apoptosis, Mtb-infected cells were stained with Annexin V and propidium iodide (PI). Flow cytometry experiments demonstrated that there was no difference in the percentage of apoptotic cells between BNIP3-KO and control macrophages in response to Mtb infection (Fig. 5A), indicating that BNIP3 does not play a role in the induction of apoptosis in Mtb-infected macrophages. A recent report suggested that increased mitophagy in *Mycobacterium bovis* infection inhibits xenophagy [39], and in this study, the reduction of mitophagy by BNIP3 KO tended to be accompanied by an increase in xenophagy, but the difference was not significant (Additional file 1: Fig. S5). Next, we evaluated the intracellular survival of Mtb in BNIP3 knockdown (siBNIP3) or BNIP3-KO macrophages to investigate the role of BNIP3 deficiency in the survival of mycobacteria. Our results demonstrated that the number of colony-forming units (CFU) was significantly decreased at each time point in Mtb-infected BNIP3-KO macrophages compared to the control group (Fig. 5B, Additional file 1: Fig. S6). Similarly, the number of CFU was lower in Mtb-infected siBNIP3 cells than in the siControl group (Fig. 5C). Because mROS production was significantly increased in BNIP3-KO macrophages during Mtb infection (Fig. 3D), we hypothesized that higher mROS levels were responsible for decreased Mtb survival. To test this hypothesis, the cells were treated with MitoTEMPO, an mROS scavenger. As expected, the reduction of mROS levels increased the number of CFU compared to the control group (Fig. 5E). Furthermore, the intracellular survival ratio of Mtb was increased in WT RAW 264.7 cells treated with MitoTEMPO compared to the controls (Fig. 5D). These results indicate that the inhibition of mitophagy through the downregulation of BNIP3 increased mROS production, resulting in reduced Mtb survival.

**Fig. 6:**
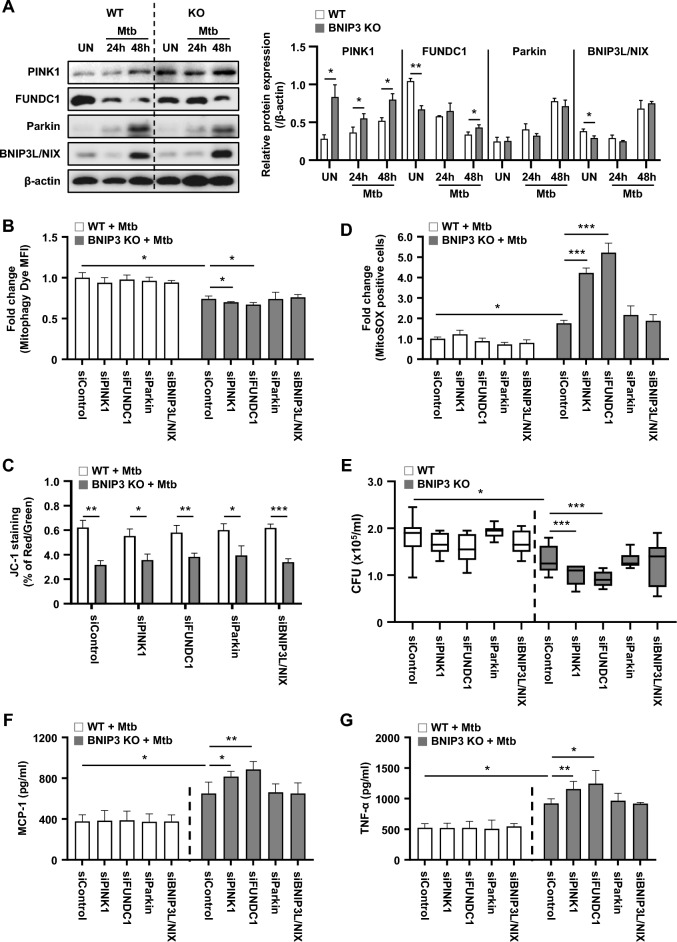
Mitophagy receptors regulate Mtb. **A** RAW 264.7 cells were infected with Mtb (MOI = 1) for 24 and 48 h. **B**–**G** WT and BNIP3-KO RAW 264.7 cells were transfected with siRNA and then infected with Mtb (MOI = 1) for 48 h. **B** Mitophagy was measured in RAW 264.7 cells using the mitophagy detection kit (*n* = 4 per group). **C** MMP was measured in RAW 264.7 cells using JC-1 staining. Fluorescent intensity of JC-1 was detected at excitation wavelength 488 nm and emission wavelength 530 nm using flow cytometry (*n* = 4 per group). **D** WT and BNIP3-KO RAW 264.7 cells were incubated with MitoSOX Red to measure mROS levels. The mROS levels were analyzed using flow cytometry (*n* = 4 per group). **E** Intracellular survival was assayed by enumerating the CFU (*n* = 9 per group). **F**, **G** The analysis of proinflammatory cytokine levels was performed in macrophages at 48 h post infection (*n* = 6 per group). The data are presented as means ± SD of at least three independent experiments; *p < 0.05, **p < 0.01, ***p < 0.001

Next, we checked the effects of other mitophagy molecules, such as Parkin, BNIP3L/NIX, PTEN-induced kinase 1 (PINK1), and FUN14 domain containing 1 (FUNDC1), in WT and BNIP3-KO macrophages. The expression of Parkin and BNIP3L/NIX was increased in Mtb-infected macrophages, but there was no difference between the WT and BNIP3-KO macrophages (Fig. 6A). In contrast, PINK1 and FUNDC were increased in BNIP3-KO macrophages compared to the WT during Mtb infection at 24 and 48 h (Fig. 6A). In addition, levels of mitophagy were significantly decreased in siPINK1 and siFUNDC1-transfected BNIP3-KO macrophages compared to the siControl group during Mtb infection (Fig. 6B), suggesting that BNIP3 plays an important role in mitophagy induction.

We then examined mROS levels in both WT and BNIP3-KO macrophages after Mtb infection, and the mROS level significantly increased in BNIP3-KO macrophages transfected with siPINK1 and siFUNDC1 compared to the siControl group (Fig. 6D). The MMP of all small interfering RNA (siRNA)-transfected KO cells was significantly reduced compared to the WT controls. However, there were no differences in MMP among siControl-transfected KO cells and other siRNA-transfected KO cells (Fig. 6C). Interestingly, the intracellular survival of Mtb was significantly decreased in siPINK1 and siFUNDC1-transfected BNIP3-KO macrophages compared to the siControl group (Fig. 6E). The levels of MCP-1 and TNF-α were also elevated in siPINK1 and siFUNDC1-transfected BNIP3-KO RAW 264.7 cells compared to the siControls at 48 h during Mtb infection (Fig. 6F, G). These results suggest that both PINK1 and FUNDC1 supplement mitophagy to inhibit the production of mROS in BNIP3-KO cells following Mtb infection.

### BNIP3 is not related to autophagy induction in Mtb-infected macrophages

**Fig. 7:**
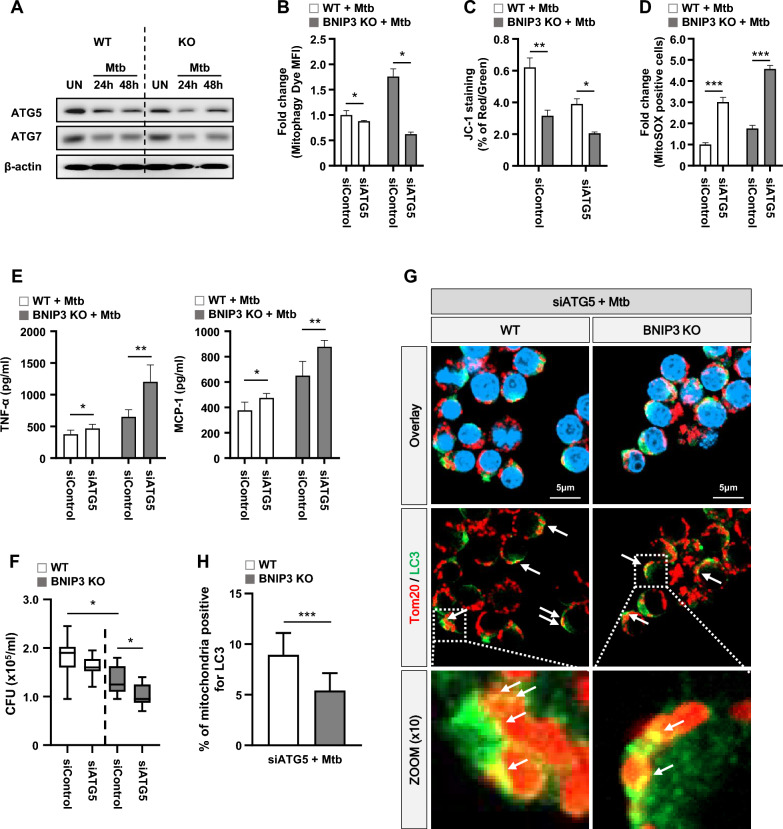
The autophagy-related gene ATG5 controls mitophagy. **A** RAW 264.7 cells were infected with Mtb (MOI = 1) for 24 and 48 h. **B**–**H** WT and BNIP3-KO RAW 264.7 cells were transfected with siRNA and then infected with Mtb (MOI = 1) for 48 h. **B** Mitophagy was measured in RAW 264.7 cells using a mitophagy detection kit (*n* = 4 per group). **C** MMP was measured in RAW 264.7 cells using JC-1 staining. Fluorescent intensity of JC-1 was detected at excitation wavelength 488 nm and emission wavelength 530 nm using flow cytometry (*n* = 4 per group). **D** WT and BNIP3-KO RAW 264.7 cells were incubated with MitoSOX Red to measure mROS levels. The mROS levels were analyzed using flow cytometry (*n* = 4 per group). **E** The analysis of proinflammatory cytokine levels was performed in macrophages at 48 h post infection (*n* = 6 per group). **F** Intracellular survival was assayed by enumerating the CFU (*n* = 9 per group). **G** WT and BNIP3-KO RAW 264.7 cells were transfected with siRNA and then infected with Mtb (MOI = 1) for 48 h, stained with LC3 (green), Tom20 (red), and DAPI (blue), and visualized using confocal microscopy. **H** Quantification data in the Fig. 7G. Quantification data of LC3 recruitment to mitochondria were collected from 50 to 60 cells from at four independent experiments. The data are presented as means ± SD of at least three independent experiments; *p < 0.05, **p < 0.01 ***p < 0.001

To determine whether BNIP3 deficiency affects autophagy, we analyzed autophagy machinery proteins, including Unc-51 like autophagy activating kinase (ULK) 1, autophagy related gene (ATG)14, and WD repeat domain phosphoinositide-interacting protein (WIPI) 2, in Mtb-infected macrophages. During Mtb infection, the recruitment of proteins in autophagic machinery to mitochondria was found to be similar between WT and BNIP3 KO macrophages (Additional file 1: Fig. S7). The expression levels of ATG5 and ATG7 also were not different between the WT and BNIP3-KO macrophages (Fig. 7A). Next, we investigated the role of ATG5, an important protein in autophagy, in mitophagy. The knockdown of ATG5 reduced the levels of mitophagy in the BNIP3-KO RAW 264.7 cells during Mtb infection (Fig. 7B). In siATG5-transfected BNIP3-KO RAW 264.7 cells, Mtb significantly reduced MMP compared to the WT controls (Fig. 7C). In addition, the levels of mROS and proinflammatory cytokines (TNFα and MCP-1) were increased in siATG5-transfected BNIP3-KO RAW 264.7 cells during Mtb infection (Fig. 7D, E). We hypothesized that the increased levels of mROS and proinflammatory cytokines due to the knockdown of ATG5 inhibited the intracellular survival of Mtb, and we confirmed the viability of Mtb. As expected, intracellular Mtb was reduced in the siATG5-transfected BNIP3-KO RAW 264.7 cells compared to the siControl (Fig. 7F). Moreover, knockdown of ATG5 clearly reduced the recruitment of LC3 to damaged mitochondria in BNIP3-KO RAW 264.7 cells compared to the WT controls (Fig. 7G, H). These results suggest that a deficiency of ATG5 in BNIP3-KO cells strongly decreases mitophagy, leading to the induction of damaged mitochondria. As a result, the elevation of mROS and cytokines, which inhibit intracellular Mtb, are induced in BNIP3-KO cells. Thus, we propose that BNIP3-induced mitophagy is not related to autophagy induction, but that the inhibition of autophagy can result in a suppression of mitophagy.

**Fig. 8:**
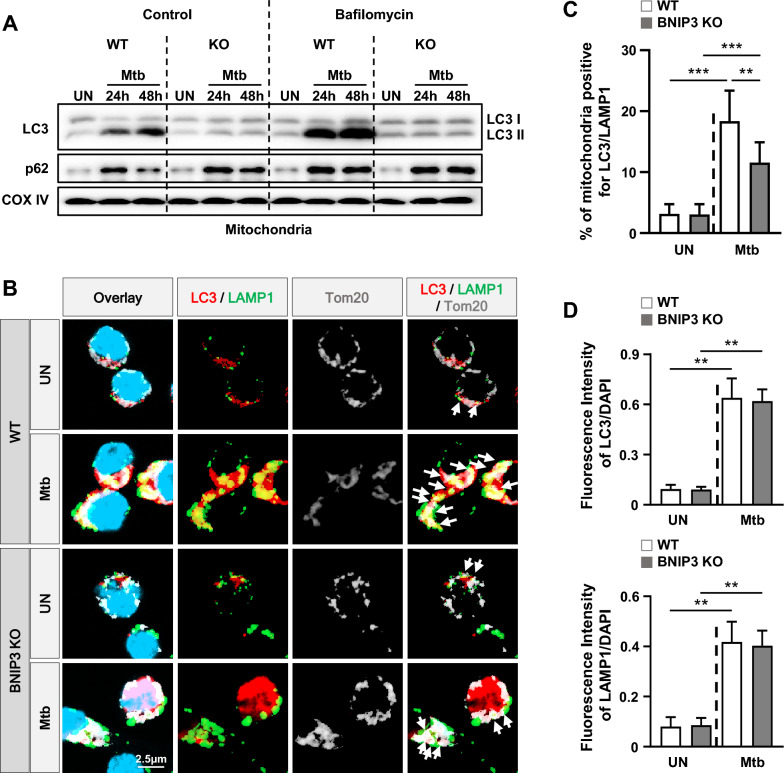
BNIP3 regulates the translocation of LC3 to mitochondria. **A** RAW 264.7 cells were treated with bafilomycin (100 nM) for 1 h and then infected with Mtb at an MOI of 1 for 48 h. Western blot analysis was performed using mitochondrial fractions from Mtb-infected macrophages. **B** RAW 264.7 cells were infected with Mtb for 48 h, and then stained with LC3 (red), LAMP1 (green), Tom20 (gray), and DAPI (blue). **C** Quantification data for LC3 and LAMP1 recruitment to mitochondria were collected from 100 cells from at five independent experiments. Quantification data in the **B**. **D** Ratio of LC3 and LAMP1 fluorescence intensity was observed in B (*n* = 5 per group). The data are presented as means ± SD of at least three independent experiments; *p < 0.05, **p < 0.01 ***p < 0.001

Next, we observed the formation of autophagosome and lysosome in mitochondria during Mtb infection. In control, Mtb induced LC3-II and reduced p62 in WT compared to BNIP3 KO macrophages at 48h (Fig. 8A). Additionally, Mtb increased the levels of both LC3-II and p62 in bafilomycin-treated WT macrophages compared to WT controls. In contrast, bafilomycin treatment in BNIP3 KO macrophages did not induce dramatic changes as observed in WT during Mtb infection. In BNIP3 KO macrophages, Mtb decreased the recruitment of LC3 and lysosomal-associated membrane protein (LAMP) 1 to mitochondria compared to WT (Fig. 8B, C). However, the expression levels of LC3 and LAMP1 induced by Mtb were not different between WT and BNIP3-KO macrophages (Fig. 8D). These data suggest that BNIP3 deficiency inhibits autophagolysosome formation by reducing LC3 translocation to the mitochondria.

### BNIP3 is critical for regulating intracellular Mtb survival in vivo

**Fig. 9:**
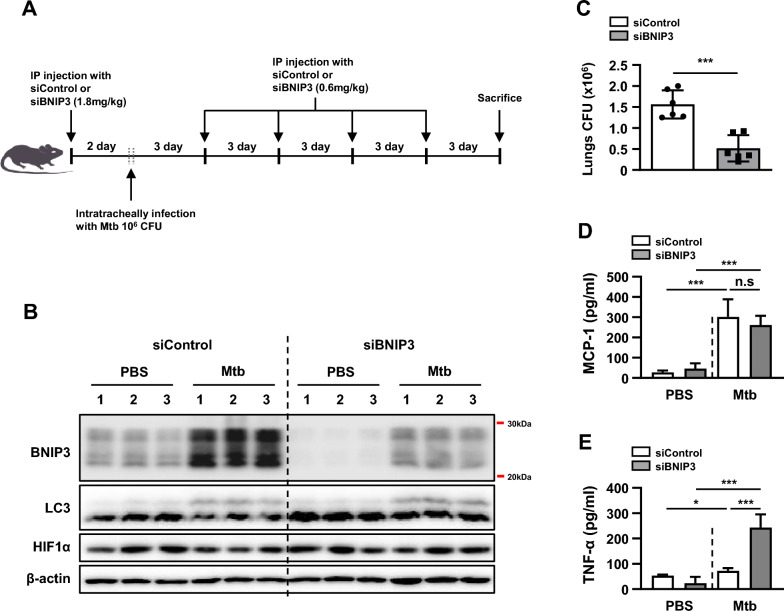
Regulation of BNIP3 controls the intracellular survival of Mtb in vivo*.* **A** C57BL/6 WT mice were intraperitoneally injected with siControl or siBINP3 (1.8 mg/kg) using the LIPID-based in vivo transfection reagent (6 mice per group). After 2 days, mice were intratracheally infected with Mtb (1 × 10^6^ CFU), and then injected with siRNA (0.6 mg/kg) for 15 consecutive days, once every 3 days. **B** The levels of BNIP3, LC3, HIF1α, and β-actin were evaluated using western blotting. **C** Bacterial burden was measured in the lungs 15 days after infection (*n* = 6 mice per group). **D, E** Levels of MCP-1 and TNF-α in the sera of Mtb-infected mice were analyzed by ELISA (*n* = 6 mice per group). The data are presented as means ± SD of at least three independent experiments; *p < 0.05, ***p < 0.001

To examine the functions of BNIP3 expression levels in Mtb-infected mice, we investigated whether siBNIP3 regulates intracellular Mtb in mice lungs. We confirmed a reduction of BNIP3 in the lung tissue of mice transfected with siBNIP3 (Fig. 9B). In Fig. 9B, we did not detect a difference in LC3-II between siBNIP3-transfected mice and control mice during Mtb. This is consistent with our previous data, in which the mitophagy induced by BNIP3 did not increase autophagy. In the BNIP3-deficient mouse model, HIF1α was not increased compared to the controls, which may not have affected HIF1a by the deficiency of BNIP3. As expected, the survival of mycobacteria in the lungs of BNIP3-knockdown mice was significantly decreased compared to the controls (Fig. 9C). Next, we analyzed proinflammatory cytokine production in the sera of control and BNIP3-knockdown mice. The Mtb infection increased the levels of MCP-1 and TNF-α in both BNIP3-knockdown and controls (Fig. 9D, E). The expression level of MCP-1 was not significantly different between the BNIP3-knockdown mice and the controls (Fig. 9D). However, the level of TNF-α was significantly elevated in Mtb-infected BNIP3-knockdown mice compared to the controls (Fig. 9E). These results are consistent with our in vitro data (Fig. 4B). These data suggest that inhibition of BNIP3 expression suppresses intracellular Mtb in the lungs of mice.

## Discussion

**Fig. 10:**
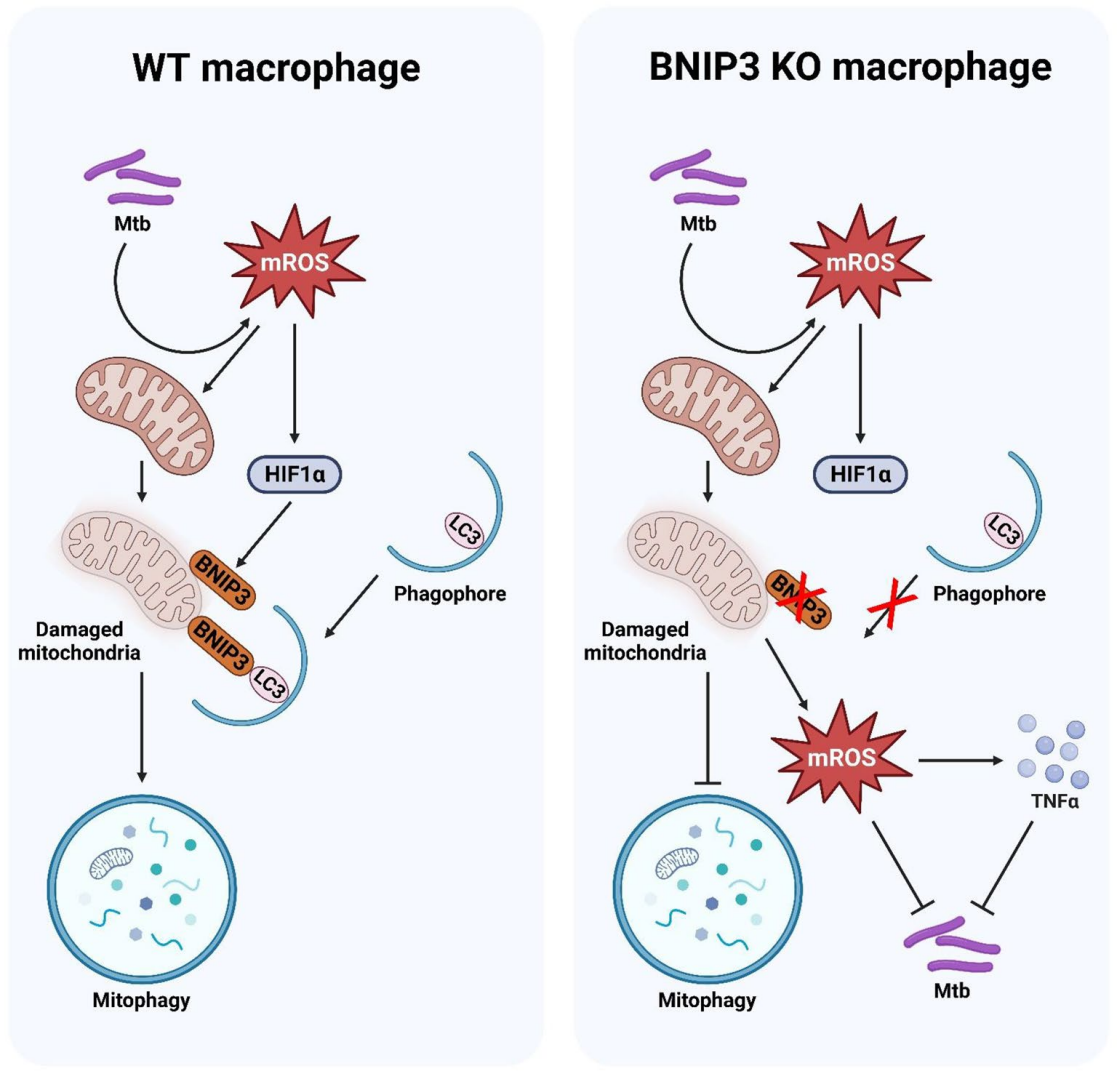
Schematic diagram. Mtb increases mROS through reduction of MMP in macrophages. Induced mROS promote the mitophagy pathway by inducing BNIP3 production through an increase in HIF1α. In contrast, mitophagy is reduced in BNIP3-deficient macrophages, resulting in elevated levels of mROS and TNF-α and inhibition of Mtb growth (Created with BioRender.com)

Mitochondria are the major source of ROS generation in mammalian cells. Excessive ROS oxidize mitochondrial DNA, proteins, and lipids, resulting in strand breaks and base modifications and leading to numerous pathologies, including cancer, neurological diseases, and cell death [40]. The accumulation of ROS in mitochondria can also trigger the release of additional ROS, further increasing mitochondrial dysfunction [41]. To maintain cellular homeostasis, damaged mitochondria are eliminated via mitophagy [42]. In this study, we demonstrated that HIF1α-BNIP3-mediated mitophagy was involved in the elimination of damaged mitochondria, resulting in a reduction of mROS and proinflammatory cytokines during Mtb infection (Fig. 10).

We observed that both ESAT6 stimulation and live Mtb infection upregulated BNIP3 protein expression; however, heat-killed Mtb infection did not affect BNIP3 expression in BMDMs. These findings suggest that live Mtb, as well as virulent Mtb antigens, can induce BNIP3 expression, leading to the activation of mitophagy. It has been established that BNIP3 is a downstream target of HIF1α [28, 29]. Our results showed that the inhibition of HIF1α reduced BNIP3 expression in macrophages during Mtb infection, confirming previous studies [28, 29]. Several groups have reported that HIF1α is activated by ROS [43–45]. Here, we showed that BNIP3 and HIF1α in Mtb-infected macrophages treated with a ROS scavenger. These data indicate that ROS elevated BNIP3 production via the upregulation of HIF1α expression. Several studies have reported that under hypoxic stress, BNIP3 can induce apoptosis through the recruitment of BAX and BAK in host mitochondria [37, 38]. Although BNIP3 is a BH3-only protein that can induce apoptosis, various normal cells can trigger mitophagy without inducing apoptosis [31, 32, 38]. In NK cells, BNIP3 plays a role in the elimination of damaged mitochondria during mouse cytomegalovirus (MCMV) infection [46]. Our results showed that BNIP3 did not induce apoptosis; however, it triggered mitophagy in response to Mtb infection. Furthermore, the activation of mitophagy was reduced in BNIP3-KO macrophages compared to WT macrophages, suggesting that Mtb-induced BNIP3 could be beneficial for Mtb survival in macrophages via mitophagy induction.

Mitochondrial dysfunction due to oxidative stress can initiate the autophagy pathway known as mitophagy [13]. In mitophagy, many molecules are involved, but we observed mitophagy induced by BNIP3 in macrophages infected with Mtb. It is difficult to explain why BNIP3 KO induces other mitophagy-related proteins such as PINK1 and FUNDC1. However, our data suggest that BNIP3 might be a key molecule inducing mitophagy in Mtb-infected macrophages. ATG5 is important in autophagy as it is involved in the formation of the autophagosome membrane and autophagosome-lysosome fusion [47]. We found that the reduction of mitophagy by BNIP3 deficiency did not affect autophagy; however, inhibition of autophagy by siATG5 decreased mitophagy. Taken together, autophagy molecules might be critical for mitophagy induction.

In this study, we showed that the accumulation of defective mitochondria due to BNIP3 deficiency significantly increased the mROS levels during Mtb infection. The levels of proinflammatory cytokines, such as TNF-α and MCP-1, were also elevated in the BNIP3-KO macrophages compared to those in the control group. However, MCP-1 was not increased in BNIP3-deficient mice, which was probably due to differences in in vitro and in vivo conditions. Although further study is necessary to determine the function of BNIP3 in cytokine production following mycobacterial infection, BNIP3 plays an important role in proinflammatory cytokine synthesis.

Previous reports have indicated that the accumulation of mROS is caused by mitochondrial damage due to reduced MMP [6, 35]. Therefore, mitophagy is necessary to remove damaged mitochondria to maintain mitochondrial homeostasis in cells, since the accumulation of damaged mitochondria induces the continuous production of mROS [11, 12, 35]. Another group reported that the inhibition of BNIP3 expression elevates mROS levels due to the accumulation of defective mitochondria in the liver [48], supporting our findings. Thus, BNIP3 expression levels play an important role in the regulation of mROS synthesis in macrophages.

Although the importance of mROS during Mtb infection is not well understood, our results show that mROS play key roles in the inhibition of Mtb via proinflammatory cytokine production. It was previously reported that induced mROS play a role in the induction of host defense responses, such as antimicrobial signaling, inflammasome activation, and proinflammatory cytokine production [49–51]. It is well known that proinflammatory cytokines regulate the growth, differentiation, and activation of immune cells to control intracellular pathogens [52]. In this study, we showed increased levels of TNF-α in the sera of BNIP3-deficient mice during Mtb infection. Many reports suggest that TNF-α is a key factor in phagocyte activation, since TNF-deficient mice fail to form granulomas due to a severe deficiency in the recruitment of immune cells in the lungs during Mtb infection [53, 54]. Another study suggested that the accumulation of mROS by mitophagy inhibition triggers TNF-α production [55]. Thus, we suggest that BNIP3 deficiency induces mROS accumulation, leading to an elevation of TNF-α levels. Several studies have proposed that the production of ROS and proinflammatory cytokines is essential for host resistance to Mtb infection [49, 56, 57]. Here, we showed that BNIP3-induced mROS suppressed the intracellular survival of Mtb through proinflammatory cytokine production. This is the first study to demonstrate the role of BNIP3 in mROS production in Mtb-infected macrophages.

## Conclusion

In this study, we showed that BNIP3 activates mitophagy in Mtb-infected macrophages. In these macrophages, BNIP3-induced mitophagy is responsible for the regulation of ROS and proinflammatory cytokine levels, suggesting that BNIP3 plays an important role in controlling intracellular Mtb. These findings indicate that BNIP3 could be a potential therapeutic target for the treatment of tuberculosis.

## Materials and methods

### Cell culture

BMDMs were isolated from C57BL6 mice and differentiated for 4 days in Dulbecco’s minimal essential medium (DMEM; Welgene) containing 25 ng/ml macrophage colony-stimulating factor (M-CSF; R&D Systems) and supplemented with 10% fetal bovine serum (FBS; Welgene), 100 IU/ml penicillin (Welgene), and 100 μg/ml streptomycin (Welgene). All animal experiments were performed in accordance with Korean Food and Drug Administration guidelines. The murine macrophage cell line RAW 264.7 (American Type Culture Collection; ATCC) was cultured in DMEM supplemented with 10% FBS, 100 IU/ml penicillin, and 100 mg/ml streptomycin. The cell cultures were maintained at 5% CO_2_ and 37 °C in polypropylene tissue culture plates (Corning).

### Mtb culture and infection

Mtb H37Rv strain (Mtb; ATCC 27294) was cultured in Middlebrook 7H9 liquid medium (BD Biosciences) supplemented with 10% OADC (oleic acid, albumin, dextrose, and catalase) and 5% glycerol. Bacteria were suspended in phosphate-buffered saline (PBS; Welgene) at a concentration of 1 × 10^8^ CFU/ml. Mtb was stored at − 80 °C. The cells were infected with heat-killed or live Mtb at an MOI of 1:1 or 1:5 for 3 h. To remove non-infected bacteria, the cells were washed and cultured in a medium containing 5% FBS. Heat-killed Mtb was prepared by heating live Mtb in PBS at 80 °C for 30 min. To measure the survival of intracellular Mtb, BMDMs and RAW 264.7 cells were infected with Mtb and lysed in distilled water to allow the collection of intracellular bacteria. The lysates were plated separately on 7H10 agar (BD Biosciences) plates, and incubated at 37 °C for 2–3 weeks. Colony counting was performed in triplicate.

### Measurement of ROS

Intracellular ROS or mROS levels were measured using a dihydroethidium (DHE) or MitoSox red staining assay. RAW 264.7 cells were infected with Mtb and incubated with DHE (20 μM) or MitoSox red (5 μM) for 30 min at 37 °C in 5% CO_2_. The samples were analyzed using a FACSCanto II cytometer (BD Biosciences). Data were processed using FlowJo software (Tree Star).

### Polymerase chain reaction (PCR)

Total RNA was isolated from Mtb-infected BMDMs or RAW 264.7 cells, and mRNA was reverse transcribed into cDNA using TRIzol reagent (Invitrogen) according to the manufacturer’s instructions. Reverse transcription-PCR was performed using Prime Taq Premix (Genet Bio) to detect the mRNA levels of the target genes. For quantitative real-time PCR, cDNA was synthesized, and target gene expression was quantified using SYBR green (QIAGEN). The mean values of the triplicate reactions were normalized to the mean value of β-actin. The sequences of the primers used were as follows: mouse *Bnip3* (forward: 5′-GCTCCTGGGTAGAACTGCAC-3′, reverse: 5′-GCTGGGCATCCAACAGTATT-3′) and mouse *Actb* (forward: 5′-CCACCATGTACCCAGGCATT-3′, reverse: 5′-AGGGTGTAAAACGCAGCTCA-3′).

### Western blot analysis

Mtb-infected cells were lysed in a radioimmunoprecipitation assay buffer (ELPIS, Daejeon, South Korea) containing a protease inhibitor cocktail. The extracted proteins were separated on an SDS-PAGE gel before being transferred to a polyvinylidene difluoride membrane (Millipore, Billerica, MA, USA). Next, the membranes were probed with primary antibodies against BNIP3, HIF1α, LC3, β-actin (Cell Signaling Technology, Danvers, MA, USA), COX IV, and anti-β-tubulin (Abcam, Cambridge, MA, USA), followed by the secondary antibodies anti-rabbit IgG-HRP (Cell Signaling Technology) and anti-mouse IgG-HRP (Calbiochem, Darmstadt, Germany). The membranes were developed using a chemiluminescence reagent (Millipore) and quantified using an Alliance Mini imaging system (UVItec Cambridge, UK). The ROS scavenger NAC and the HIF1α inhibitor FM19G11 were purchased from Sigma-Aldrich.

### Establishment of transgenic RAW 264.7 cell lines

To delete *Bnip3* in RAW 264.7 cells, we selected three guide RNAs targeting the *Bnip3* region that were designed using the web design tool (https://chopchop.cbu.uib.no/). Selected guide RNAs were cloned into a px330 vector and validated in RAW 264.7 cells by western blot analysis. Validated guide RNAs (target sites: 5′-GCTGAAGTGCAGTTCTACCC-3′), along with Cas9 mRNA, were introduced by Lipofectamine 3000 (Invitrogen) into RAW 264.7 cells. The resulting gDNAs were screened for deletions in the *Bnip3* region with primers flanking *Bnip3* (forward: 5′- CCACAAGTGGTCAGATTGCTAA-3′, reverse: 5′-CTTGGAGCTACTTCGTCCAGAT -3′) by PCR and Sanger sequencing. CRISPR/Cas9 protocol was performed as described previously [58].

### Transfection of siRNA

Silencing of *Bnip3, PINK1, FUNDC1, Parkin, BNIP3L/NIX*, and *ATG5* was performed using siRNAs (100 nM) targeting mouse *Bnip3* (Bioneer), *PINK1, FUNDC1, Parkin, BNIP3L/NIX*, and *ATG5* mRNA sequences (Santa Cruz Biotechnology) and negative control siRNAs (Bioneer). The siRNA oligonucleotides were transfected into cultured BMDMs using Lipofectamine 3000 (Invitrogen) according to the manufacturer’s instructions.

### Apoptosis analysis

Cell death was assessed using an Annexin V/PI staining kit according to the manufacturer’s instructions (BD Biosciences). Briefly, the cells were stained with FITC-conjugated Annexin V and PI, and flow cytometry was performed using a FACSCanto II with FACS Diva. The results were analyzed using FlowJo software (BD Biosciences).

### Mitochondria morphology analysis

Mitochondrial fractions from macrophages were obtained using the Mitochondria Isolation Kit for Cultured Cells (Thermo Scientific) according to the manufacturer’s instructions. The MMP assay was performed using 5,5′,6,6′-tetrachloro-1,1′,3,3′-tetraethylbenzimidazolycarbocyanine iodide (JC-1; Invitrogen). RAW 264.7 cells were stained with JC-1 (2 μM) for 30 min at 37 °C in 5% CO_2_. Mitophagy was assessed using a Mitophagy Detection Kit (Dojindo Molecular Technologies). Cells were treated according to the manufacturer’s protocol, and samples were analyzed on a FACSCanto II cytometer (BD Biosciences). Data were processed using FlowJo software (Tree Star).

### Enzyme-linked immunosorbent assay (ELISA)

A sandwich ELISA kit (BD Pharmingen) was used to measure the levels of secreted cytokines, including MCP-1 and TNF-α, in the culture supernatants. Assays were performed according to the manufacturer’s instructions. Triplicate samples were analyzed using an ELISA reader (Molecular Devices), and concentrations were calculated using a standard curve. MitoTEMPO was purchased from Sigma-Aldrich.

### Mouse infection in vivo

All animal procedures were reviewed and approved by the Institutional Animal Care and Use Committee of Chungnam National University (CNU-00907). Delivery of siRNA in vivo was intraperitoneally injected with negative control siRNA or mouse BNIP3 siRNA (Bioneer) following the instructions of the LIPID-based in vivo transfection reagent kit (Altogen Biosystems). Female C57BL/6 WT mice (6 weeks of age) were first injected with siRNA (1.8 mg/kg) and then infected with Mtb on day 2. Next, mice were injected with siRNA (0.6 mg/kg) on 15 consecutive days, once every 3 days. Mice were intratracheally infected with Mtb (1 × 10^6^ CFU) in 50 μl PBS. The control mice were injected with an equal volume of PBS (Fig. 8A). Lung cells and sera were harvested from individual mice groups at 15 days after intratracheal infection.

### Immunofluorescence

RAW 264.7 cells were grown on 18-mm coverslips for overnight. The cells were fixed in 4% paraformaldehyde and then washed three times with PBS. The cells were cultured with primary antibodies overnight, and then with the appropriate secondary antibody (Alexa Fluor 594 anti-mouse IgG and Alexa Fluor 488 anti-rabbit IgG, Life Technologies) for 2 h at room temperature. Next, the cells were stained with DAPI to label DNA. The stained cells were visualized under a LSM 900 confocal microscope (Zeiss).

### Statistical analysis

Data are shown as means ± SD, and all experiments were performed at least three times. The results of the experiments were evaluated using Student’s t-test or one-way analysis of variance (ANOVA), followed by Bonferroni’s multiple comparison tests. Statistical significance between groups was determined using the Mann–Whitney and Kruskal–Wallis tests. Statistical significance is indicated by *p < 0.05, ** *p* < 0.01, and *** *p* < 0.001.

### Supplementary Information

**Additional file 1:** **Figure S1.** Mtb regulates mitophagy in macrophage. Original recordings of Mtphagy Dye fluorescence detection by flow cytometry (BD FACSCANTO II) was performed using 488 nm for excitation and 695 nm for emission reflecting the mitophagy in Figure 3B. WT and BNIP3-KO RAW 264.7 cells were infected with Mtb for 3 h. The cells were analyzed 24 and 48 h post infection. **Figure S2.** Lysosomal inhibitors decrease Mtb-induced mitophagy in macrophages. RAW 264.7 cells were treated with bafilomycin (100 nM) or chloroquine (50 μM) for 1 h and then infected with Mtb at an MOI of 1 for 48 h. (mean ± SD of n = 3). **Figure S3.** Mtb reduces mitochondrial DNA in macrophages. RAW 264.7 cells were infected with Mtb at an MOI of 1 for 48 h. And then cells were immunostained with α-DNA antibody (green) and Tom20 (red). (mean ± SD of n = 8). **Figure S4.** Mitochondrial proteins suppress by Mtb in macrophages. WT and BNIP3-KO RAW 264.7 cells were infected with Mtb for 48 h, and then stained with anti-Tom20 or anti-Tim23 antibody (green) and DAPI (blue). **Figure S5.** BNIP3 can control xenophagy in macrophages. WT and BNIP3-KO RAW 264.7 cells were infected with Mtb expressing red fluorescent protein at MOI = 1 for 48 h and were then immunostained using anti-LC3 antibody (green) and DAPI (blue). Colocalization of Mtb with LC3 were counted in total of 100 bacterial cells. Results are representatives from at least three independent experiments (mean ± SD of n = 5). **Figure S6.** BNIP3 regulates intracellular Mtb in macrophages. RAW 264.7 cells were infected with Mtb and then lysed using distilled water. Drops of 20 μl of 10−1 to 10−3 dilutions of Mtb and derivatives were spotted onto Middlebrook 7H10-OADC agar plates. **Figure S7.** BNIP3 is not related recruitment of autophagy molecules to mitochondria. RAW 264.7 cells were infected with Mtb at MOI = 1 for 48 h and were then immunostained. Results are representatives from at least three independent experiments (mean ± SD of n = 5). **Figure S8.** The effect of specific siRNA. RAW 264.7 cells were transfected with each siRNA, and then incubated with 48 h.

## Data Availability

The data that support the findings of this study are available in the Material and Methods section of this article.

## Abbreviations

Mtb: *Mycobacterium tuberculosis*
TB: Tuberculosis
ROS: Reactive oxygen species
MOI: Multiplicity of infection
CFU: Colony-forming units
M-CSF: Macrophage colony-stimulating factor
NAC: N-acetyl cysteine
BMDMs: Bone marrow-derived macrophages
TNF: Tumor necrosis factor
LPS: Lipopolysaccharide
PBS: Phosphate-buffered saline
MCP: Monocyte chemoattractant protein
CoCl2: Cobalt(II) chloride
BNIP3: BCL2/adenovirus E1B 19 kDa protein-interacting protein 3
MMP: Mitochondrial membrane potential
LC3: Microtubule-associated protein 1A/1B-light chain 3
DENV: Dengue virus
HBV: Human hepatitis B virus
*L. monocytogenes*: *Listeria monocytogenes*
ESAT-6: Early secretory antigenic target 6 kDa
HIF1α: Hypoxia-inducible factor-1-alpha
LIR: LC3-interacting region
MCMV: Mouse cytomegalovirus

## References

[1] Sia JK, Rengarajan J, Fischetti VA, Novick RP, Ferretti JJ, Portnoy DA, Braunstein M, Rood JI. Immunology of *Mycobacterium tuberculosis* infections. Microbiol Spectr. 2019. 10.1128/microbiolspec.GPP3-0022-2018. 10.1128/microbiolspec.gpp3-0022-2018 PMC6636855 31298204

[2] Cambier CJ, Falkow S, Ramakrishnan L (2014). Host evasion and exploitation schemes of *Mycobacterium tuberculosis*. Cell.

[3] Pieters J (2008). *Mycobacterium tuberculosis* and the macrophage: maintaining a balance. Cell Host Microbe.

[4] Kaufmann SHE, Dorhoi A (2016). Molecular determinants in phagocyte-bacteria interactions. Immunity.

[5] Mohareer K, Medikonda J, Vadankula GR, Banerjee S (2020). Mycobacterial control of host mitochondria: bioenergetic and metabolic changes shaping cell fate and infection outcome. Front Cell Infect Microbiol.

[6] Tiku V, Tan M-W, Dikic I (2020). Mitochondrial functions in infection and immunity. Trends Cell Biol.

[7] Lee J, Song C-H (2021). Effect of reactive oxygen species on the endoplasmic reticulum and mitochondria during intracellular pathogen infection of mammalian cells. Antioxidants.

[8] Hamacher-Brady A, Brady NR (2016). Mitophagy programs: mechanisms and physiological implications of mitochondrial targeting by autophagy. Cell Mol Life Sci.

[9] Wang L, Qi H, Tang Y, Shen HM (2020). Post-translational modifications of key machinery in the control of mitophagy. Trends Biochem Sci.

[10] Wang L, Lu G, Shen HM (2020). The long and the short of PTEN in the regulation of mitophagy. Front Cell Dev Biol.

[11] Gkikas I, Palikaras K, Tavernarakis N (2018). The role of mitophagy in innate immunity. Front Immunol.

[12] Palikaras K, Lionaki E, Tavernarakis N (2018). Mechanisms of mitophagy in cellular homeostasis, physiology and pathology. Nat Cell Biol.

[13] Zhang J (2013). Autophagy and mitophagy in cellular damage control. Redox Biol.

[14] Quinsay MN, Thomas RL, Lee Y, Gustafsson ÅB (2010). Bnip3-mediated mitochondrial autophagy is independent of the mitochondrial permeability transition pore. Autophagy.

[15] Merjaneh M, Langlois A, Larochelle S, Cloutier CB, Ricard-Blum S, Moulin VJ (2017). Pro-angiogenic capacities of microvesicles produced by skin wound myofibroblasts. Angiogenesis.

[16] Zhou H, Du W, Li Y, Shi C, Hu N, Ma S, Wang W, Ren J (2018). Effects of melatonin on fatty liver disease: the role of NR4A1/DNA-PKcs/p53 pathway, mitochondrial fission, and mitophagy. J Pineal Res.

[17] Zhou H, Yue Y, Wang J, Ma Q, Chen Y (2018). Melatonin therapy for diabetic cardiomyopathy: a mechanism involving Syk-mitochondrial complex I-SERCA pathway. Cell Signal.

[18] Barbier V, Lang D, Valois S, Rothman AL, Medin CL (2017). Dengue virus induces mitochondrial elongation through impairment of Drp1-triggered mitochondrial fission. Virology.

[19] Kim SJ, Khan M, Quan J, Till A, Subramani S, Siddiqui A (2013). Hepatitis B virus disrupts mitochondrial dynamics: induces fission and mitophagy to attenuate apoptosis. PLoS Pathog.

[20] Liu H, You L, Wu J, Zhao M, Guo R, Zhang H, Su R, Mao Q, Deng D, Hao Y (2020). Berberine suppresses influenza virus-triggered NLRP3 inflammasome activation in macrophages by inducing mitophagy and decreasing mitochondrial ROS. J Leukoc Biol.

[21] Stavru F, Bouillaud F, Sartori A, Ricquier D, Cossart P (2011). Listeria monocytogenes transiently alters mitochondrial dynamics during infection. Proc Natl Acad Sci USA.

[22] Zhang Y, Yao Y, Qiu X, Wang G, Hu Z, Chen S, Wu Z, Yuan N, Gao H, Wang J (2019). Listeria hijacks host mitophagy through a novel mitophagy receptor to evade killing. Nat Immunol.

[23] Sun LL, Shao YN, You MX, Li CH (2022). ROS-mediated BNIP3-dependent mitophagy promotes coelomocyte survival in Apostichopus japonicus in response to Vibrio splendidus infection. Zool Res.

[24] Lee J, Choi JA, Cho SN, Son SH, Song CH (2019). Mitofusin 2-deficiency suppresses *Mycobacterium tuberculosis* survival in macrophages. Cells.

[25] Choi HH, Shin DM, Kang G, Kim KH, Park JB, Hur GM, Lee HM, Lim YJ, Park JK, Jo EK (2010). Endoplasmic reticulum stress response is involved in *Mycobacterium tuberculosis* protein ESAT-6-mediated apoptosis. FEBS Lett.

[26] Lim YJ, Choi JA, Lee JH, Choi CH, Kim HJ, Song CH (2015). Mycobacterium tuberculosis 38-kDa antigen induces endoplasmic reticulum stress-mediated apoptosis via toll-like receptor 2/4. Apoptosis.

[27] Jamwal S, Midha MK, Verma HN, Basu A, Rao KV, Manivel V (2013). Characterizing virulence-specific perturbations in the mitochondrial function of macrophages infected with *Mycobacterium tuberculosis*. Sci Rep.

[28] Daskalaki I, Gkikas I, Tavernarakis N (2018). Hypoxia and selective autophagy in cancer development and therapy. Front Cell Dev Biol.

[29] Fu Z-J, Wang Z-Y, Xu L, Chen X-H, Li X-X, Liao W-T, Ma H-K, Jiang M-D, Xu T-T, Xu J (2020). HIF-1α-BNIP3-mediated mitophagy in tubular cells protects against renal ischemia/reperfusion injury. Redox Biol.

[30] Bonello S, Zähringer C, BelAiba RS, Djordjevic T, Hess J, Michiels C, Kietzmann T, Görlach A (2007). Reactive oxygen species activate the HIF-1alpha promoter via a functional NFkappaB site. Arterioscler Thromb Vasc Biol.

[31] Hanna RA, Quinsay MN, Orogo AM, Giang K, Rikka S, Gustafsson ÅB (2012). Microtubule-associated protein 1 light chain 3 (LC3) interacts with Bnip3 protein to selectively remove endoplasmic reticulum and mitochondria via autophagy*. J Biol Chem.

[32] Zhu Y, Massen S, Terenzio M, Lang V, Chen-Lindner S, Eils R, Novak I, Dikic I, Hamacher-Brady A, Brady NR (2013). Modulation of serines 17 and 24 in the LC3-interacting region of Bnip3 determines pro-survival mitophagy versus apoptosis*. J Biol Chem.

[33] Iwashita H, Torii S, Nagahora N, Ishiyama M, Shioji K, Sasamoto K, Shimizu S, Okuma K (2017). Live cell imaging of mitochondrial autophagy with a novel fluorescent small molecule. ACS Chem Biol.

[34] Lazarou M, Sliter DA, Kane LA, Sarraf SA, Wang C, Burman JL, Sideris DP, Fogel AI, Youle RJ (2015). The ubiquitin kinase PINK1 recruits autophagy receptors to induce mitophagy. Nature.

[35] Held NM, Houtkooper RH (2015). Mitochondrial quality control pathways as determinants of metabolic health. BioEssays.

[36] Forrester SJ, Kikuchi DS, Hernandes MS, Xu Q, Griendling KK (2018). Reactive oxygen species in metabolic and inflammatory signaling. Circ Res.

[37] Kubli DA, Ycaza JE, Gustafsson AB (2007). Bnip3 mediates mitochondrial dysfunction and cell death through Bax and Bak. Biochem J.

[38] Zhang J, Ney PA (2009). Role of BNIP3 and NIX in cell death, autophagy, and mitophagy. Cell Death Differ.

[39] Song Y, Ge X, Chen Y, Hussain T, Liang Z, Dong Y, Wang Y, Tang C, Zhou X (2022). Mycobacterium bovis induces mitophagy to suppress host xenophagy for its intracellular survival. Autophagy.

[40] Bjelland S, Seeberg E (2003). Mutagenicity, toxicity and repair of DNA base damage induced by oxidation. Mutat Res.

[41] Suzuki K, Kirisako T, Kamada Y, Mizushima N, Noda T, Ohsumi Y (2001). The pre-autophagosomal structure organized by concerted functions of APG genes is essential for autophagosome formation. Embo j.

[42] Chen G, Kroemer G, Kepp O (2020). Mitophagy: an emerging role in aging and age-associated diseases. Front Cell Dev Biol.

[43] Gao A, Jiang J, Xie F, Chen L (2020). Bnip3 in mitophagy: novel insights and potential therapeutic target for diseases of secondary mitochondrial dysfunction. Clin Chim Acta.

[44] Belaidi E, Morand J, Gras E, Pépin J-L, Godin-Ribuot D (2016). Targeting the ROS-HIF-1-endothelin axis as a therapeutic approach for the treatment of obstructive sleep apnea-related cardiovascular complications. Pharmacol Ther.

[45] Manuelli V, Pecorari C, Filomeni G, Zito E (2022). Regulation of redox signaling in HIF-1-dependent tumor angiogenesis. FEBS J.

[46] O’Sullivan Timothy E, Johnson Lexus R, Kang Helen H, Sun Joseph C (2015). BNIP3- and BNIP3L-mediated mitophagy promotes the generation of natural killer cell memory. Immunity.

[47] Ye X, Zhou X-J, Zhang H (2018). Exploring the role of autophagy-related gene 5 (ATG5) yields important insights into autophagy in autoimmune/autoinflammatory diseases. Front Immunol.

[48] Glick D, Zhang W, Beaton M, Marsboom G, Gruber M, Simon MC, Hart J, Dorn GW, Brady MJ, Macleod KF (2012). BNip3 regulates mitochondrial function and lipid metabolism in the liver. Mol Cell Biol.

[49] Shekhova E (2020). Mitochondrial reactive oxygen species as major effectors of antimicrobial immunity. PLoS Pathog.

[50] Rimessi A, Previati M, Nigro F, Wieckowski MR, Pinton P (2016). Mitochondrial reactive oxygen species and inflammation: molecular mechanisms, diseases and promising therapies. Int J Biochem Cell Biol.

[51] Hulsmans M, Van Dooren E, Holvoet P (2012). Mitochondrial reactive oxygen species and risk of atherosclerosis. Curr Atheroscler Rep.

[52] Turner MD, Nedjai B, Hurst T, Pennington DJ (2014). Cytokines and chemokines: At the crossroads of cell signalling and inflammatory disease. Biochim Biophys Acta.

[53] Roach DR, Bean AGD, Demangel C, France MP, Briscoe H, Britton WJ (2002). TNF regulates chemokine induction essential for cell recruitment, granuloma formation, and clearance of mycobacterial infection. J Immunol.

[54] Cooper AM, Mayer-Barber KD, Sher A (2011). Role of innate cytokines in mycobacterial infection. Mucosal Immunol.

[55] Xu Y, Shen J, Ran Z (2020). Emerging views of mitophagy in immunity and autoimmune diseases. Autophagy.

[56] Flynn JL, Goldstein MM, Chan J, Triebold KJ, Pfeffer K, Lowenstein CJ, Schreiber R, Mak TW, Bloom BR (1995). Tumor necrosis factor-alpha is required in the protective immune response against Mycobacterium tuberculosis in mice. Immunity.

[57] Di Paolo NC, Shafiani S, Day T, Papayannopoulou T, Russell DW, Iwakura Y, Sherman D, Urdahl K, Shayakhmetov DM (2015). Interdependence between Interleukin-1 and tumor necrosis factor regulates TNF-dependent control of mycobacterium tuberculosis infection. Immunity.

[58] Ran FA, Hsu PD, Wright J, Agarwala V, Scott DA, Zhang F (2013). Genome engineering using the CRISPR-Cas9 system. Nat Protoc.

