# Supplementary material for: Impaired mitophagy induces antimicrobial responses in macrophages infected with Mycobacterium tuberculosis
Source: Cell Biosci. 2023 Aug 30;13:158. doi: 10.1186/s13578-023-01107-2 (PMC10470153; doi:10.1186/s13578-023-01107-2)
Supplement: Supplementary file 1 — Additional file 1: Figure S1. Mtb regulates mitophagy in macrophage. Original recordings of Mtphagy Dye fluorescence detection by flow cytometry (BD FACSCANTO II) was performed using 488 nm for excitation and 695 nm for emission reflecting the mitophagy in Figure 3B. WT and BNIP3-KO RAW 264.7 cells were infected with Mtb for 3 h. The cells were analyzed 24 and 48 h post infection. Figure S2. Lysosomal inhibitors decrease Mtb-induced mitophagy in macrophages. RAW 264.7 cells were treated with bafilomycin (100 nM) or chloroquine (50 μM) for 1 h and then infected with Mtb at an MOI of 1 for 48 h. (mean ± SD of n = 3). Figure S3. Mtb reduces mitochondrial DNA in macrophages. RAW 264.7 cells were infected with Mtb at an MOI of 1 for 48 h. And then cells were immunostained with α-DNA antibody (green) and Tom20 (red). (mean ± SD of n = 8). Figure S4. Mitochondrial proteins suppress by Mtb in macrophages. WT and BNIP3-KO RAW 264.7 cells were infected with Mtb for 48 h, and then stained with anti-Tom20 or anti-Tim23 antibody (green) and DAPI (blue). Figure S5. BNIP3 can control xenophagy in macrophages. WT and BNIP3-KO RAW 264.7 cells were infected with Mtb expressing red fluorescent protein at MOI = 1 for 48 h and were then immunostained using anti-LC3 antibody (green) and DAPI (blue). Colocalization of Mtb with LC3 were counted in total of 100 bacterial cells. Results are representatives from at least three independent experiments (mean ± SD of n = 5). Figure S6. BNIP3 regulates intracellular Mtb in macrophages. RAW 264.7 cells were infected with Mtb and then lysed using distilled water. Drops of 20 μl of 10−1 to 10−3 dilutions of Mtb and derivatives were spotted onto Middlebrook 7H10-OADC agar plates. Figure S7. BNIP3 is not related recruitment of autophagy molecules to mitochondria. RAW 264.7 cells were infected with Mtb at MOI = 1 for 48 h and were then immunostained. Results are representatives from at least three independent experiments (mean ± SD of n = 5). Figure S8 [file 13578_2023_1107_MOESM1_ESM.docx]

**Additional file 1:**

**
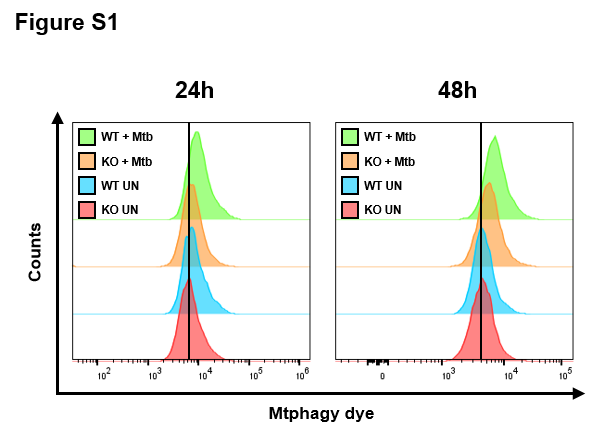
**

**Figure S1. Mtb regulates mitophagy in macrophage.** Original recordings of Mtphagy Dye fluorescence detection by flow cytometry (BD FACSCANTO II) was performed using 488 nm for excitation and 695 nm for emission reflecting the mitophagy in Figure 3B. WT and BNIP3-KO RAW 264.7 cells were infected with Mtb for 3 h. The cells were analyzed 24 and 48 h post infection.


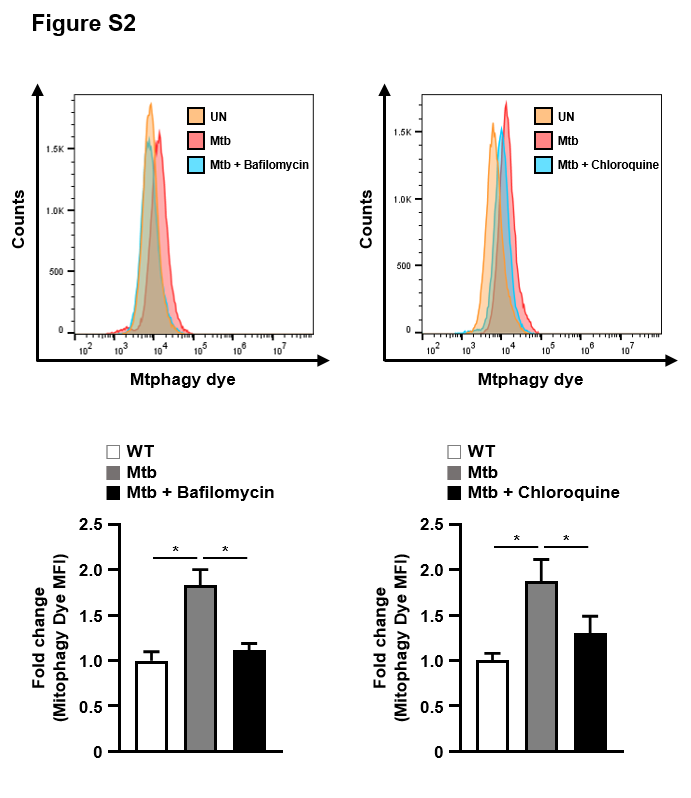


**Figure S2. Lysosomal inhibitors decrease Mtb-induced mitophagy in macrophages.** RAW 264.7 cells were treated with bafilomycin (100 nM) or chloroquine (50 μM) for 1 h and then infected with Mtb at an MOI of 1 for 48 h. (mean ± SD of *n* = 3).


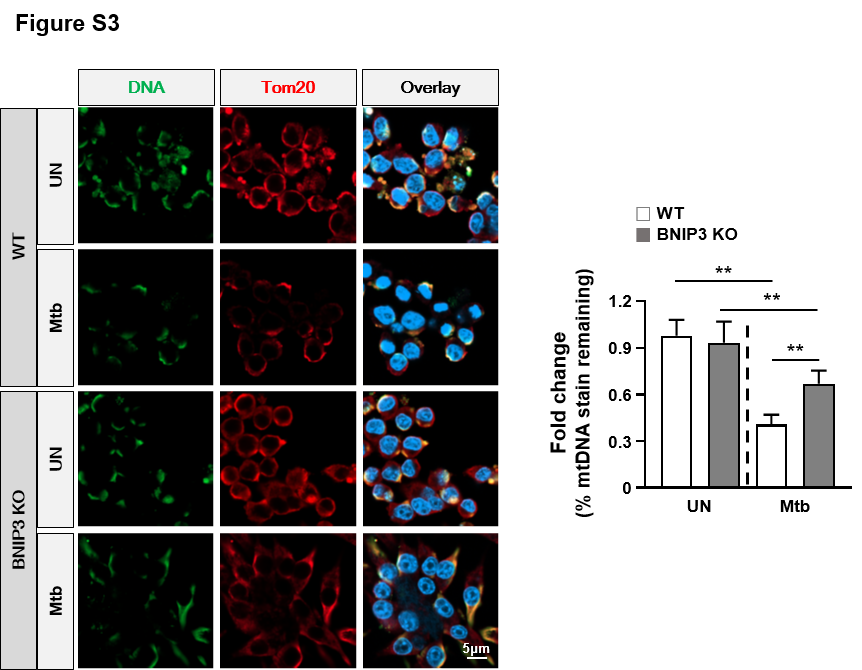


**Figure S3. Mtb reduces mitochondrial DNA in macrophages.** RAW 264.7 cells were infected with Mtb at an MOI of 1 for 48 h. And then cells were immunostained with α-DNA antibody (green) and Tom20 (red). (mean ± SD of *n* = 8).


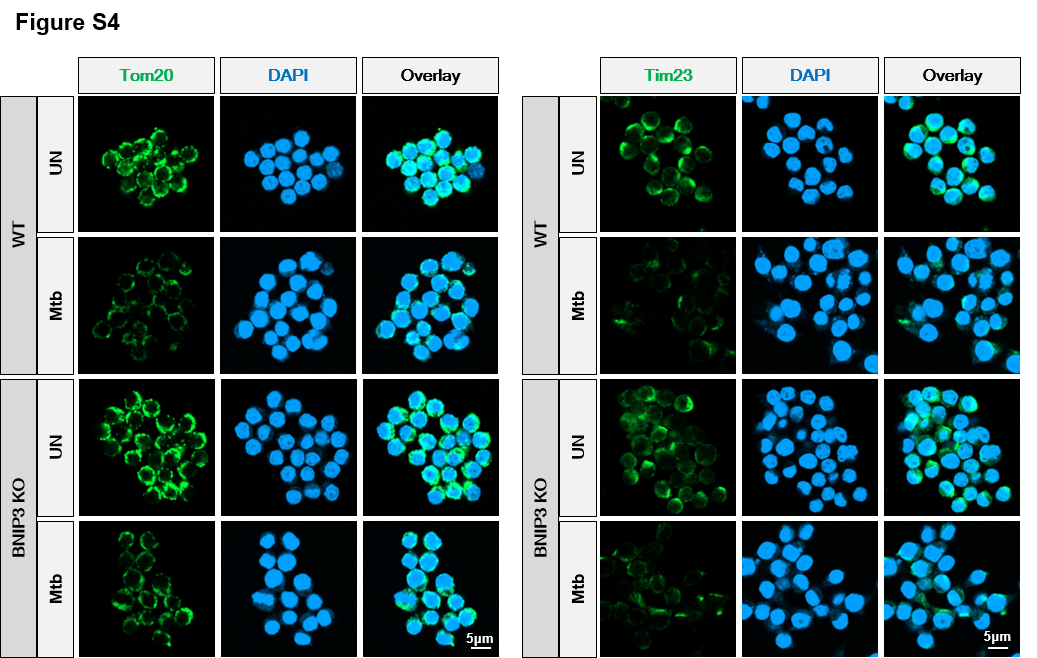


**Figure S4. Mitochondrial proteins suppress by Mtb in macrophages.** WT and BNIP3-KO RAW 264.7 cells were infected with Mtb for 48 h, and then stained with anti-Tom20 or anti-Tim23 antibody (green) and DAPI (blue).

**
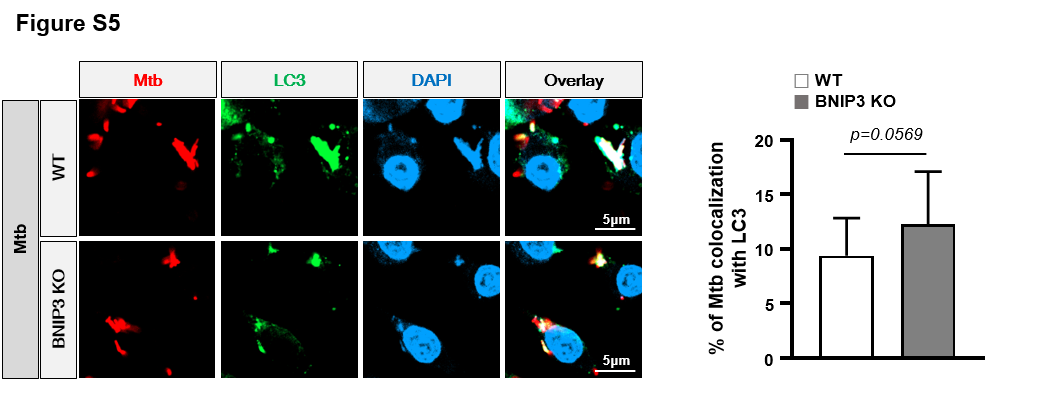
**

**Figure S5. BNIP3 can control xenophagy in macrophages.** WT and BNIP3-KO RAW 264.7 cells were infected with Mtb expressing red fluorescent protein at MOI = 1 for 48 h and were then immunostained using anti-LC3 antibody (green) and DAPI (blue). Colocalization of Mtb with LC3 were counted in total of 100 bacterial cells. Results are representatives from at least three independent experiments (mean ± SD of *n* = 5).


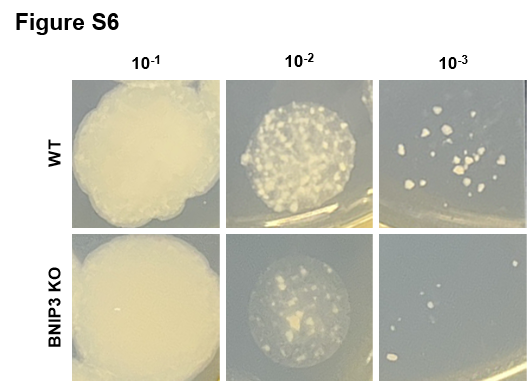


**Figure S6. BNIP3 regulates intracellular Mtb in macrophages.** RAW 264.7 cells were infected with Mtb and then lysed using distilled water. Drops of 20 μl of 10^−1^ to 10^−3^ dilutions of Mtb and derivatives were spotted onto Middlebrook 7H10-OADC agar plates.


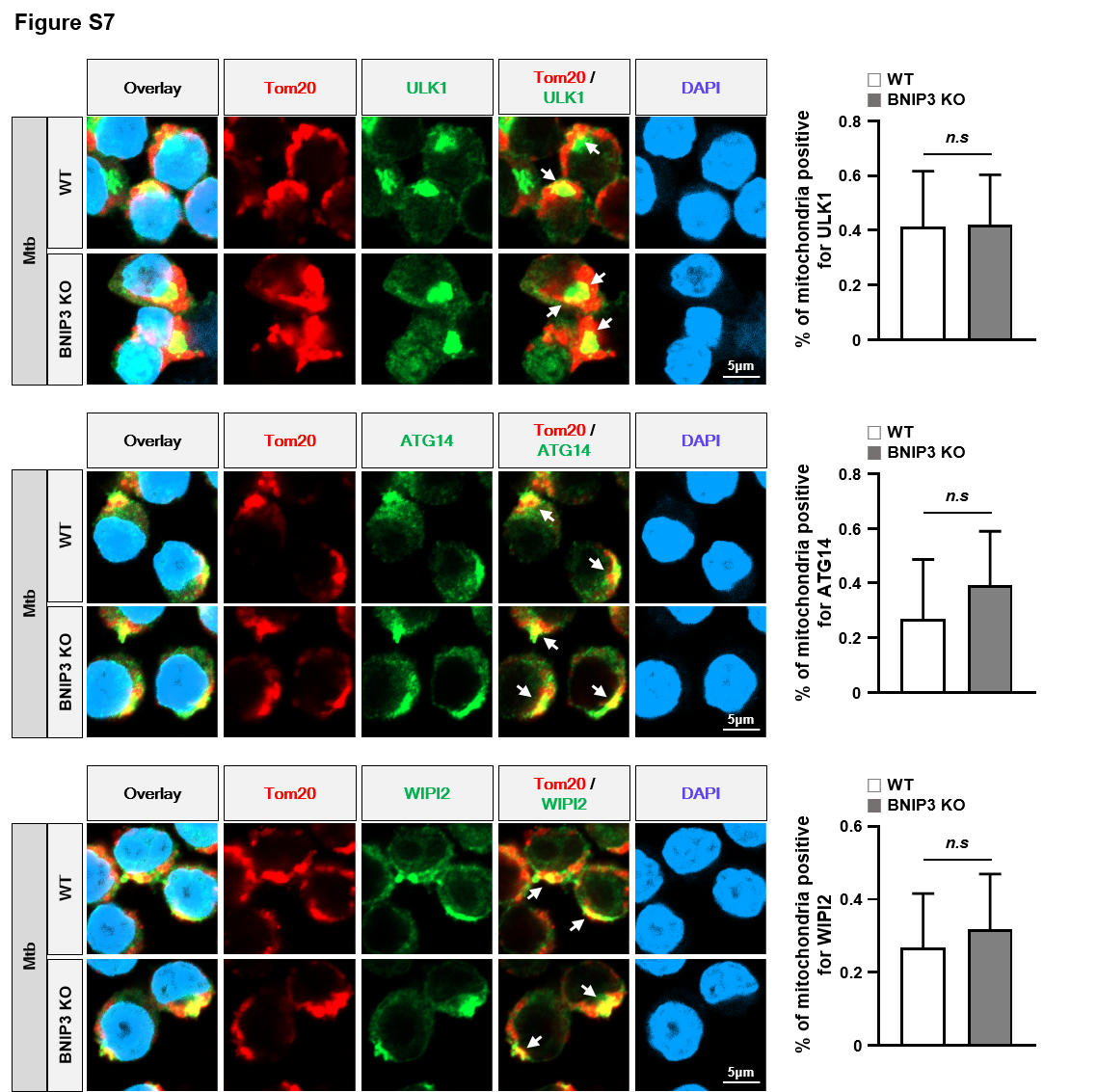


**Figure S7. BNIP3 is not related recruitment of autophagy molecules to mitochondria.** RAW 264.7 cells were infected with Mtb at MOI = 1 for 48 h and were then immunostained. Results are representatives from at least three independent experiments (mean ± SD of *n* = 5).


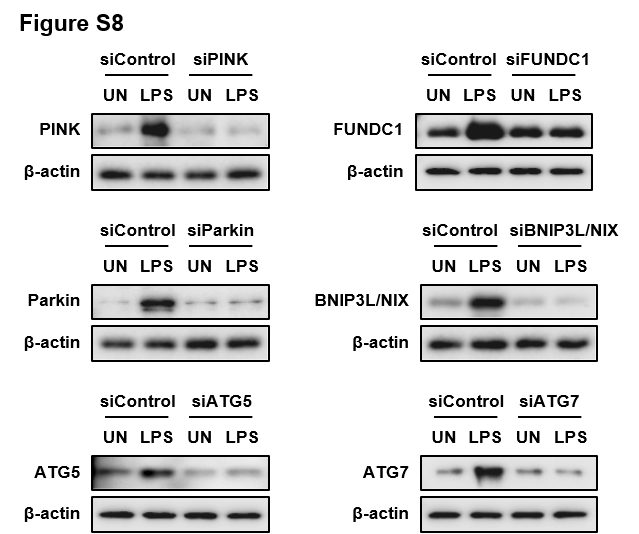


**Figure S8. The effect of specific siRNA.** RAW 264.7 cells were transfected with each siRNA, and then incubated with 48 h.
